# Assistive technologies for improving the oral hygiene of leprosy patients residing in a former leprosy colony in Betim, Minas Gerais, Brazil

**DOI:** 10.1371/journal.pone.0200503

**Published:** 2018-07-25

**Authors:** Raquel Conceição Ferreira, Marco Tulio de Freitas Ribeiro, Fabiana Vargas-Ferreira, Aline Araujo Sampaio, Ana Cristina Marinho Pereira, Andrea Maria Duarte Vargas, Rafaella Mendes de Jesus, Efigênia Ferreira e Ferreira

**Affiliations:** 1 Department of Social and Preventive Dentistry, School of Dentistry, Universidade Federal de Minas Gerais, Belo Horizonte, Brazil; 2 Casa de Saúde Santa Isabel, Fundação Hospitalar do Estado de Minas Gerais, Betim, Brazil; Berner Fachhochschule, SWITZERLAND

## Abstract

**Background:**

This study deals with management of a group of elderly patients with a history of leprosy and hand deformities by a multidisciplinary team of dentists and occupational therapists. Assistive technology devices have been developed to allow such patients to obtain independence in oral self-care and can be a cost-effective approach to improving oral care in this population. The objective of this study was to describe the development of assistive devices to facilitate daily oral hygiene in older people with enduring leprosy-related impairments.

**Methodology:**

Case study realized among elders with a history of leprosy residents in a former isolation colony in Betim, Minas Gerais, Brazil. The elders were evaluated for dependence on others for denture hygiene and mouthwash using the Daily Oral Hygiene Activity Index (ADOH). Those deemed partially or completely dependent on others were eligible for an intervention based on assistive technology. We adopted a personalized approach to each case, taking into account medical history, physical impairment and living environment. Six months after the intervention, the participants were assessed again using the ADOH and an unstructured interview about use of the devices.

**Principal findings:**

Assistive devices for denture hygiene and mouthwash were developed for 16 elders. These devices facilitated oral hygiene in most patients and there was no worsening in any of the cases. Patients’ report suggested they were satisfied with the devices provided.

**Conclusions:**

This study demonstrated that assistive devices can facilitate oral hygiene activities in leprosy patients. It also reinforces the importance of using a multidisciplinary team for the rehabilitation of these patients.

## Introduction

Leprosy is a chronic disease caused by Mycobacterium leprae, which if left untreated, can lead to severe disabilities as a result of the neurological impairment, that affect primarily the peripheral nerves and secondarily the skin and other organs [1]. Global efforts to reduce the prevalence of leprosy have borne fruit, with rates decreasing over recent decades [2]. However, Brazil is one of the 22 “global priority countries” and it was one of the three countries that reported >10 000 new cases of leprosy in 2016 [3]. In 2015, the World Health Organization (WHO) [4] reported that the proportion of new grade-2 disability cases was 6.7% globally, corresponding to a detection rate of 2.5 per million population [2].

In Brazil, leprosy patients were isolated as a prophylactic measure and this approach was maintained until the 1980s, when residents were directed to leave the colonies [5]. However, there are still many cured patients who are living in colonies and there are many of these communities around the world today, including 36 in Brazil [6]. These survivors are now elders and are living with the sequelae of the disease [7]. A study conducted in a former Brazilian leprosy colony found that 86.7% of the population had bone deformities, especially ankylosis of the joints and clawed hands and feet. Other Brazilian study showed that 79.8% of the elders with history of leprosy had grade-2 disabilities [8]. Other non-orthopedic complications included ciliary madarosis, saddle nose and blindness [6]. An Indonesian study of individuals with leprosy-related disabilities showed that 76.7% exhibited physical impairments, and most of the impairments were associated with the feet (47%), while 31% with the hands, and 11% with the eyes [9].

Such disabilities restrict the social life [9, 10] and quality of life of the individuals affected [9–11]. Moreover, this condition can be associated with dependence on others for daily activities, including oral hygiene. A study revealed that difficulty performing routine oral hygiene activities was the main factor related to oral health problems in patients with leprosy [12]. Oral hygiene is a self-care task crucial for the maintenance of health [13–16]. An accumulation of biofilm on dentures is a causative agent for oral infections, especially denture-related stomatitis [14, 16], and acts as a reservoir of bacteria and fungi that are implicated in the development of aspiration pneumonia [13, 14].

Measures that increase disabled people’s independence in activities of daily living can increase their social inclusion and improve their quality of life [17–20]. The Singular Therapy Project (STP) was chosen as a strategy for planning assistive technology-based interventions with for leprosy patients [21]. An assistive technology device is designed to compensate for physical deficiencies and it aims to maintain or improve functional skills of persons with disabilities [22, 23]. A major goal of the WHO’s Sustaining Leprosy Control Activities programs (2006–2010) was to provide rehabilitation services, with the priority given to preventing further functional neurological impairment and promotion and maintenance of self-care and health [24]. In spite of this goal, there has been scant research into the use of assistive devices by leprosy patients. A qualitative analysis showed that assistive devices were efficient and promoted the social inclusion of people affected by leprosy [18]. This study addresses that gap by describing the use of assistive technology devices to support oral hygiene activities in patients with history of leprosy in Betim, Minas Gerais, Brazil.

## Materials and methods

The research was ethically conducted in accordance with the Declaration of Helsinki. The study was approved by the Human Research Ethics Committee of the Federal University of Minas Gerais (protocol 05351612.8.0000.5149).

### Population and location

This study was conducted with elders, men and women, 60 years and older, living in the Casa de Saúde Santa Izabel; CSSI, a former leprosy colony located in the state of Minas Gerais, Southeastern, city of Betim, Brazil. There were 140 elderly people resident in the CSSI in July 2014.

In 1931, the Casa de Saúde Santa Izabel received individuals with leprosy, an infectious disease with no cure at this time. In 1937, the number of residents was 3.886 and, only in 1965, people have the right to get out, however, many of them have remained at the same colony. Nowadays, the Casa de Saúde Santa Izabel has a center of rehabilitation and care that supports the need of the patients, majority formed by elders. A multiprofessional health team (physiotherapy and occupational therapy, social workers and psychologists) assists the patients. The outpatient care includes other specialties, such as Dermatology, Ophthalmology, Cardiology, Orthopedics, Gynecology, Hansenology, Dentistry and Surgery [25].

### Study design

This case study was part of a project that consisted of treatment with dentures for edentulous older people who lacked dentures or needed their dentures replaced. After the dental prostheses were delivered, we observed that there was a need for further intervention to enable the patients to regain independence in oral self-care.

### Sample and inclusion criteria

Inclusion criteria were elders with 60 years of age or older, edentulous patients, users of dentures and those had total or partial dependence on others for oral hygiene. From 140 elders, the dependence on others for oral hygiene was evaluated in 74 residents; 66 residents were excluded at this stage (37 were edentulous and did not use dentures; a further 29 declined to participate this part of the study). The convenience sample included all the patients who have accepted to participate and need of the intervention.

### Data collection and intervention

Data were collected through interviews and observations of the elders in their residences. The first evaluation was performed between July 2015 to December 2015. The intervention was carried out in different periods for each elder. After intervention, the same elders were assessed again one week later, for adjusting (their advices) and they have received more information and orientation about the use of advices. The re-assessment six months after intervention were carried out between January and July (2016).

The dependence on others for oral hygiene was evaluated using the Daily Oral Hygiene Activity Index (ADOH) before and 6 months after intervention [26]. The ADOH can be used to track progressive loss of functional ability to manipulate the devices needed for oral self-care and the restoration of functional capacity in response to intervention and rehabilitation services [26]. It has been validated in Brazil [27]. Scores are based on observation of the patient performing four basic activities of oral hygiene: tooth or denture brushing, flossing, topical application of fluoride and mouthwash.

We investigated two activities in our sample of edentulous elders: denture brushing and mouthwash. The researcher first instructed the participants and then observed them performing the activities. For each activity, the participants were scored on a five-point scale as follows,: 0: The individual is able to perform the activity according to the assessment criteria without assistance or use of aids (total independent); 1: The individual needs some form of assistance to complete the activity effectively (partially dependent); 2: The individual spent 50% or more of effort to complete the task with or without limited supervision (supervision is limited to the initial preparation of devices necessary for hygiene, without physical contact) (partially dependent); 3: The individual spent less than 50% of effort to complete the task and required supervision with a helper or without physical help (being close, guiding, giving tips) (dependent) and 4: The individual was completely reliant on assistance and could not perform the tasks unaided (dependent). Participants who were able to complete an activity without assistance or use of an aid were classified as independent. Those who needed some help or did not complete the activity were classified as partially or totally dependent (score ≥ 1).

The ADOH was scored by two researchers trained by an occupational therapist. This training consisted of a four-hour theoretical session focused on leprosy and its sequelae, especially those affecting the hands and it included study about the ADOH criteria. Finally, the session included practical training by evaluating 10 participants together and discussing the criteria until a consensus was reached between the two researchers and the occupational therapist. After training, all evaluations were made by the two researchers. When they disagreed, cases were discussed until consensus could be reached; when needed, an occupational therapist was consulted.

All the elders (partially or totally dependent on others for oral hygiene) have received a personalized protocol and assistive devices for oral hygiene prepared by the Occupational Therapy and Dentistry team. The protocols were designed with the specific deformities and functional limitations of each individual in mind, as well as their living circumstances. All patients were oriented in the use of the devices. Moreover, all the advices were re-assessed for adjusting one week later. The assistive devices were assembled from materials such as lining, foam, PVC, thermomoldable plates. Thermomoldable plates were heated in hot water, then it was possible to copy the anatomical shape of the patients' hands until the device was fully adapted.

Six months after the intervention, the participants were re-evaluated using the ADOH. At the same time, they evaluated the intervention by answering the following questions: Are you using all the devices? How do you assess the use of the devices? Are you satisfied with them? How was the oral hygiene before and how is it now, after receiving the adaptations?

### Data analysis

The ADOH data were analyzed and absolute and relative frequencies of elders according to dependence on other for oral hygiene were calculated using Stata version 14.0.

### Ethical considerations

All participants provided written, informed consent to participation in advance using a form approved by the Research Ethics Committee of Federal University of Minas Gerais (CAAE: 05351612.8.0000.5149).

## Results

Of the 74 residents whose dependence on others for brushing and mouthwash was evaluated, 53 (71.6%) were classed as completely independent with respect to denture brushing, 12 (16.2%) were classed as partially dependent and nine (12.2%) as completely dependent on others. In the case of mouthwash, 63 (85.1%) were completely independent, six (8.1%) were partially dependent and five (6.8%) were completely dependent. Hand deformities were observed in 17.6% of the residents. A total of 24 individuals were classified as totally or partially dependent on other for at least one oral hygiene activity. There were 8 losses from this group (2 deaths, 5 refusals to participate in the research and one patient who abandoned use of the denture during the study). Thus, 16 patients, 11 women and 5 men, with a mean age of 79.9 years (*SD* = 8.43, range: 69 – 97) received the intervention, which consisted of assessment of needs, development of appropriate personalized assistive devices for oral hygiene and orientation in their use. The assistive devices developed for each patient and their relationship to the patient’s deformity and use are described in Table 1, which also gives baseline and post-intervention ADOH scores for brushing and use of mouthwash.

**Table 1:** Characteristics of the sample involving elders that was using assistive devices 6 months after intervention, devices supplied and performance of oral hygiene activities. Betim. Minas Gerais, Brazil.

Patient	Gender(M: male; F: female)	Age (years)	Place of residence	Deformities	Hygiene and mouthwash activity	Denture brushing (ADOH* scores: baseline/ final)	Mouthwash (ADOH* scores: baseline/ final)	Assistive devices
1	M	76	Long-term care facility	In both hands, rheumatoid arthritis. Incapable of right-hand prehension	Cleaned the dentures with his left hand without using the brush. For mouthwash, he didn’t use his hands	4/1	2/0	Universal strap and cup
2	M	81	Own home	In both hands: atrophy, contracture and amputation of fingers. Weak prehension on the right hand	Let the brush drop	2/1	0/0	Universal belt and thickening of the diameter of the handle of the brush
3	F	77	Own home	Wheelchair user; multiple deformities in both hands. Absence of left-hand prehension	Could not hold prosthesis during brushing	2/1	0/0	For her hygiene, she used a stabilization for dentures. Brush was permanently mounted in a position where it could be used to clean the denture.
4	F	76	Own home	Left hand with flexed contracture of all fingers, dorsal and palmar atrophy	She had difficulty holding the dentures with her left hand	2/1	2/0	She has adapted cup for mouthwash. Brush was permanently mounted in a position where it could be used to clean the denture.
5	F	69	Own home	Muscular atrophy, contracture, bone resorption in the hands; surgical amputation of some fingers	Had difficulty in holding the brush and the dentures	2/1	0/0	She has adapted cup for mouthwash. Brush was permanently mounted in a position where it could be used to clean the denture.
6	F	72	Own home	Deformities and lack of sensitivity in both hands. Unable to hold objects in her left hand; right-hand grip was precarious.	Difficulty using mouthwash and could not hold the brush firmly	2/1	0/0	Universal strap and adapted cup for mouthwash.
7	F	75	Residence	Clawed hands, bone resorption, some finger amputations and muscular atrophy with loss of the palm arches.	Capable of brushing. He was not in the habit of using the mouthwash	0/0	2/0	Cup suitable for mouthwash.
8	F	79	Own home	Wheelchair user. Deformities and lack of sensitivity in both hands	Independent in brushing. Had difficulty using mouthwash.	0/0	2/0	Cup adapted for mouthwash.
9	F	71	Own home	Deformities and lack of sensitivity in both hands. Little prehension in the right hand	Could not grip the brush firmly	2/1	2/0	Universal strap and adapted cup for mouthwash
10	F	82	Long-term care	Wheelchair user; lack of sensation in the hands and difficulty with grip in both hands	He could not carry the water with his hands to the mouthwash activity	0/0	2/0	Adapted bowl to reduce impact of dropping the dentures and prevent it fracturing and adapted cup for mouthwash.
11	M	77	Own home	Visually impaired. Muscular atrophy in both hands, and with toes contraction. Only capable of grasping with the right hand.	He has drunk directly from the tap without using his hands.	2/2	2/0	Bowl and adapted cup. No intervention was performed for brushing due to severe visual impairment.

*ADOH: Daily Oral Hygiene Activity Index.

Approximately 70% (n = 11; 68.8%) of the elders was using the assistive devices 6 months after intervention. The performance of denture brushing improved in 43.75% of the sample and 50% of the elders showed improve of the mouthwash. Use of the assistive devices did not cause a deterioration in performance in any of the participants (Tables 1 and 2). Three patients were classified as totally dependent for brushing (score 4) at baseline; of these one was able to brush his/her denture effectively using his/her device (score 1), one had a cognitive impairment that did not allow the participation of the patient. The other patient was unable to complete the task because she could not hold a denture in one hand and brush with other as she used one hand to lever herself out of her chair into a standing position. She said “I don’t care with my teeth. Give me something to walk because I have difficulty to walking”. Three others patients did not adhere to the intervention and two of them had a severe visual impairment that made intervention impossible or made it difficult to use an assistive device.

**Table 2:** Characteristics of the sample involving elders that was not using assistive devices 6 months after intervention, devices supplied and performance of oral hygiene activities. Betim. Minas Gerais, Brazil.

Patient	Gender (M: male; F: female)	Age (years)	Place of residence	Deformities	Hygiene and mouthwash activity	Denture brushing (ADOH* scores: baseline/ final)	Mouthwash (ADOH* scores: baseline/ final)	Assistive devices
1	F	97	Own home	No sensitivity in her hand; deformity of the right hand	Patient with cognitive impairment and caregiver dependency for hygiene of denture	4/4	4/4	Brush with thick cable.
2	F	87	Own home	In both hands. Poor grip in the left hand	Difficulty in brushing the dentures and in the mouthwash	2/2	0/0	Brush with thick cable. Cup suitable for mouthwash
3	F	74	Own home	Deformity and lack of sensitivity in both hands	He could not hold a denture in one hand and brush with the other as he used one hand to lever himself out of his chair into a standing position.	4/4	2/2	Bitehole brush and an anti-slip mat to protect the dentures if it fell from his hand.
4	M	95	Long-term care	Visually impaired; little prehension in either hand.	Difficulty in holding the brush	2/2	0/0	He uses a cup with handle to facilitate the mouthwash. Brush was permanently mounted in a position where it could be used to clean the dentures.
5	M	91	Own home	No vision in his left eye, impaired vision in the right eye. Flexion contracture in all the fingers. Weak grip in the left hand.	Could not hold the dentures with his left hand.	2/2	2/0	Adapted cup for mouthwash. Brush was permanently mounted in a position where it could be used to clean the dentures.

*ADOH: Daily Oral Hygiene Activity Index.

The users who adopted the assistive device provided for them were satisfied. They have realized that was improvements involving daily activities, such as hygiene buccal, as demonstrated by their comments, some of which are presented in Table 3.

**Table 3:** Patients’ opinions of their assistive devices.

Type of assistive device	Opinions
Brush with Universal belt (Fig 1)	“This brush is so good! I was shame with my last one!”
Cup suitable for mouthwash (Fig 2)	“It is beautiful! What a beautiful color!. Wow, I'll take this cup with me! It’s really fancy.”
Assistive dispositive to avoid the denture hitting the sink (Fig 3)	“It's good that it fits in the sink, right? And I can carry the cup of mouthwash with the brush inside from the bedroom to the bathroom.”
Stabilization device for cleaning prostheses (Fig 4)	“It held the prosthesis steadier”
Brush with thickened diameter (Fig 5)	“Oh, those fingers are missing!”. “The brush sometimes escaped”. “That’s good, right? It’s thicker and so it stays steadier!”
Brush fixed to the wall	“Before, the denture escaped and fell on the floor when I washed it!” “It’s very good, this one on the wall, much better”

**Fig 1:**
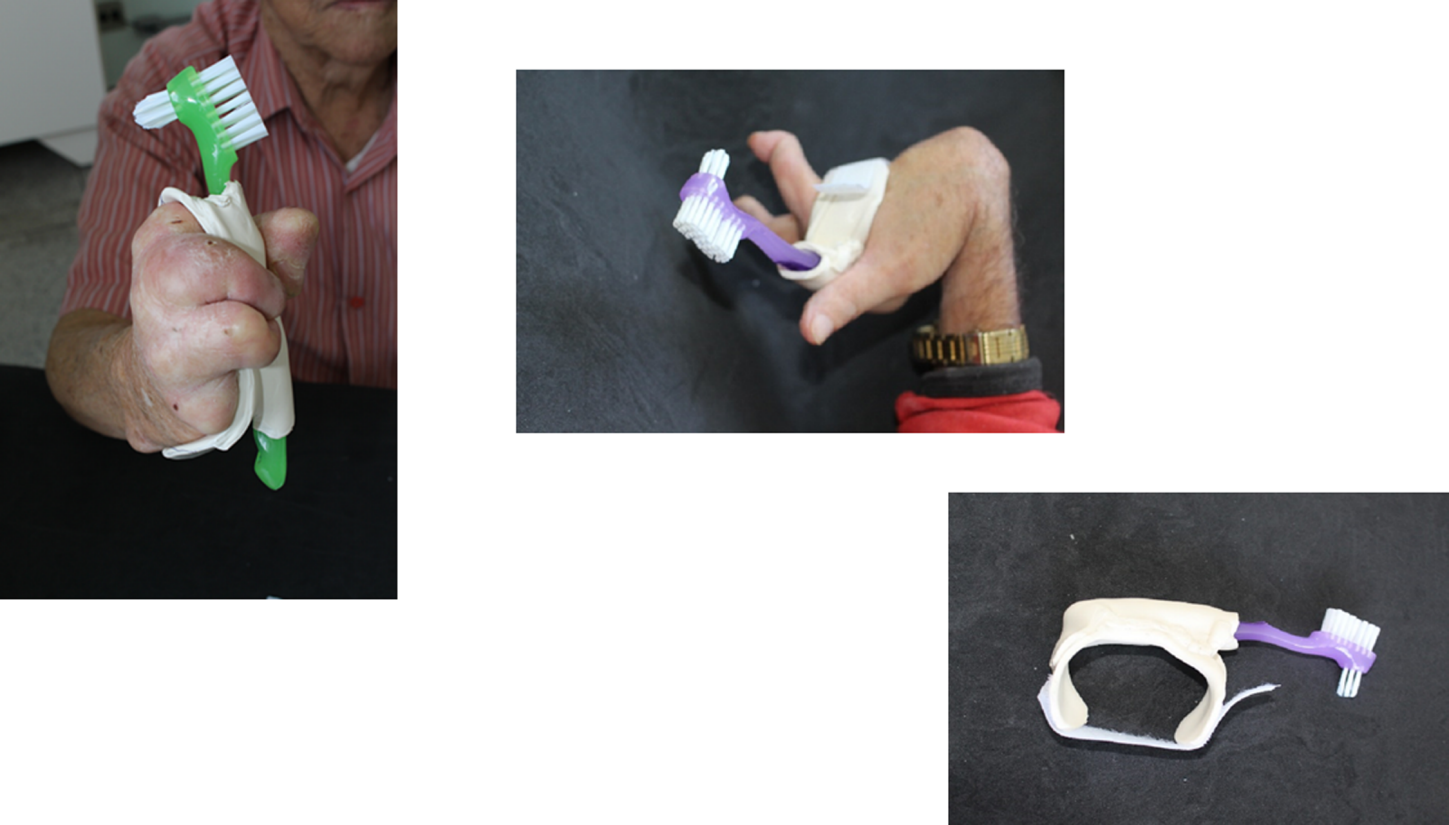
Brush with Universal belt.

**Fig 2:**
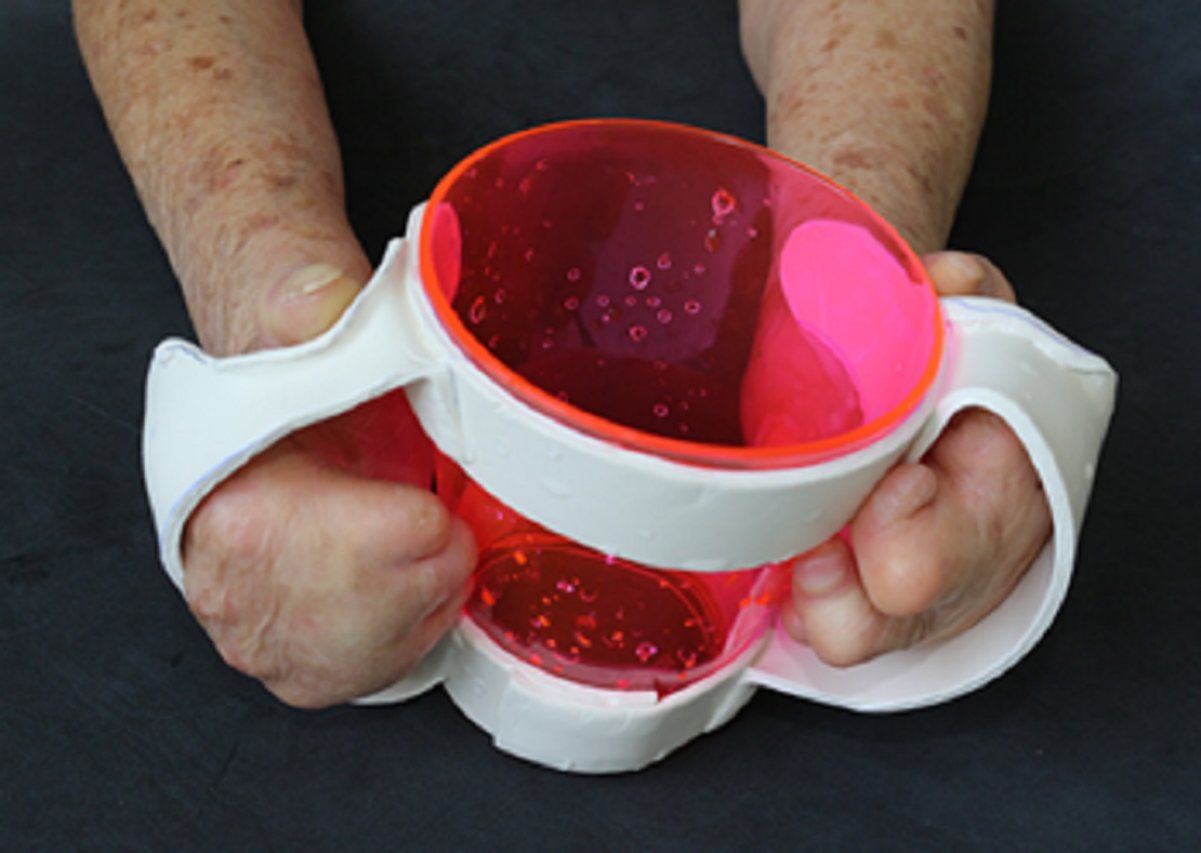
Cup suitable for mouthwash.

**Fig 3:**
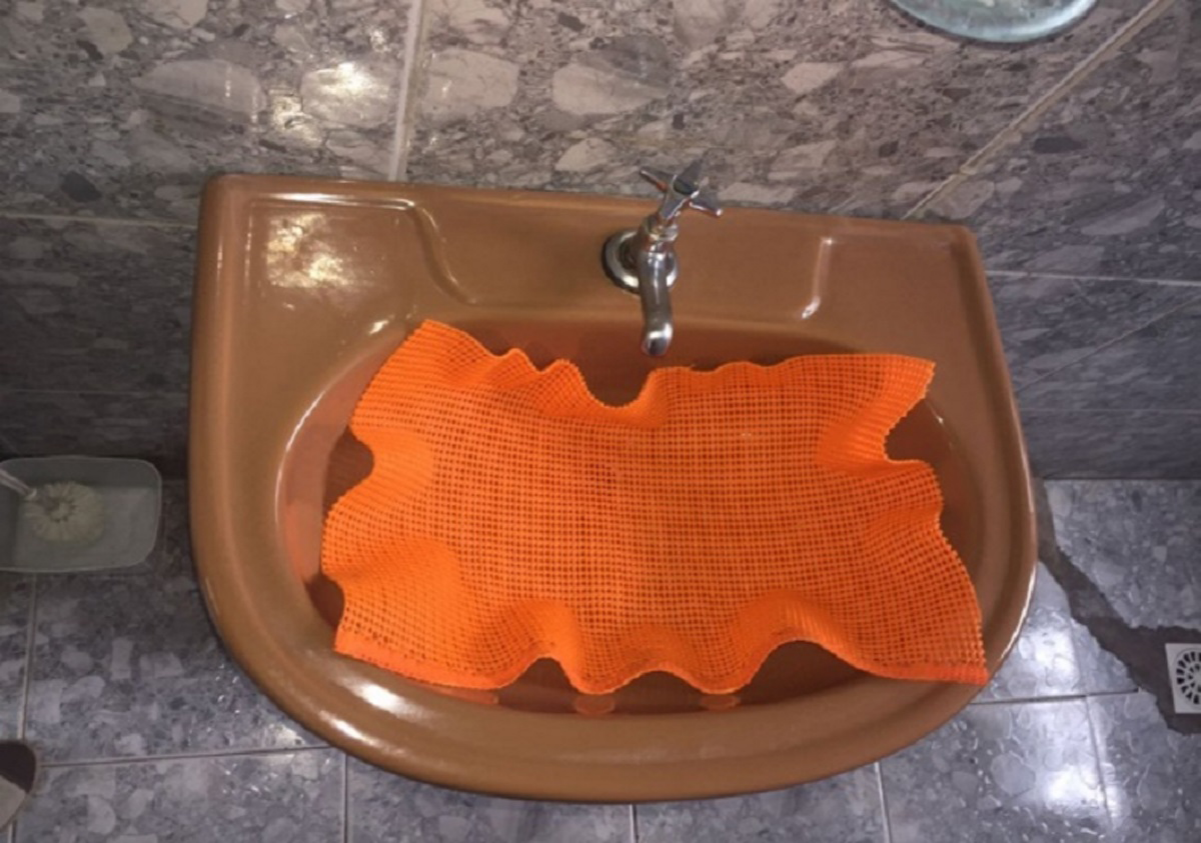
Assistive dispositive to avoid the denture hitting the sink.

**Fig 4:**
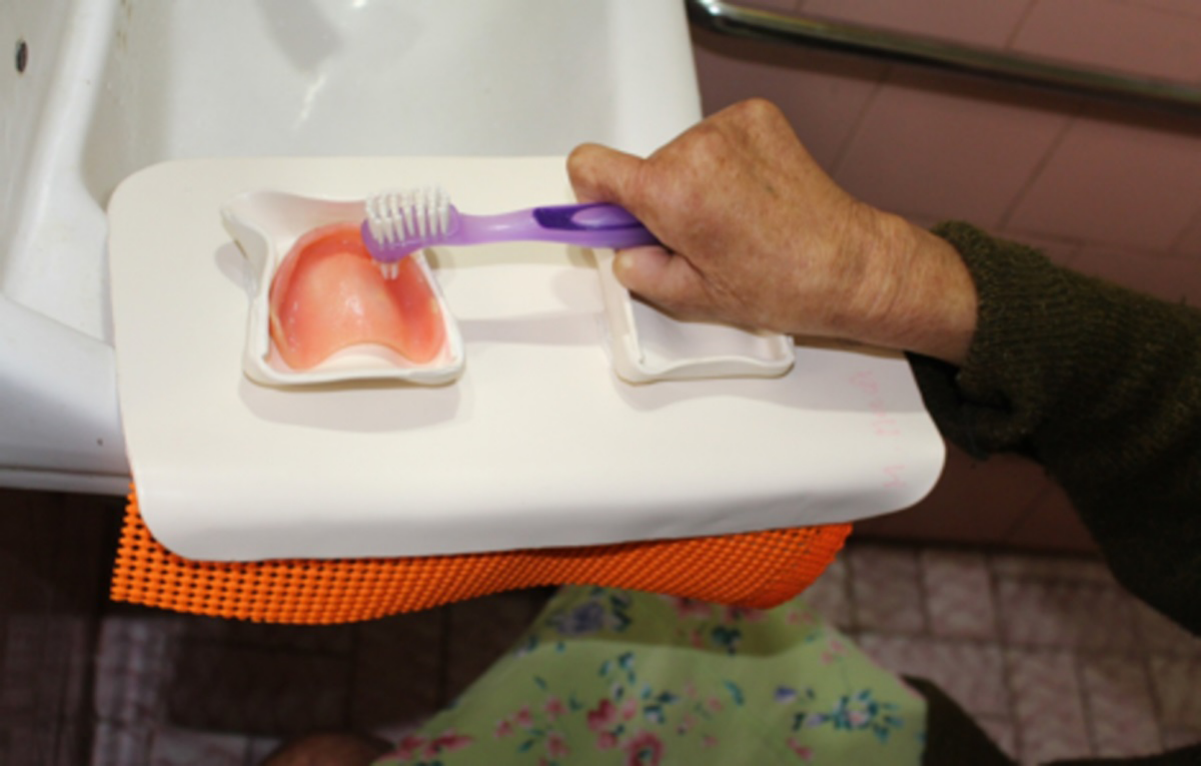
Stabilization device for cleaning dentures.

**Fig 5:**
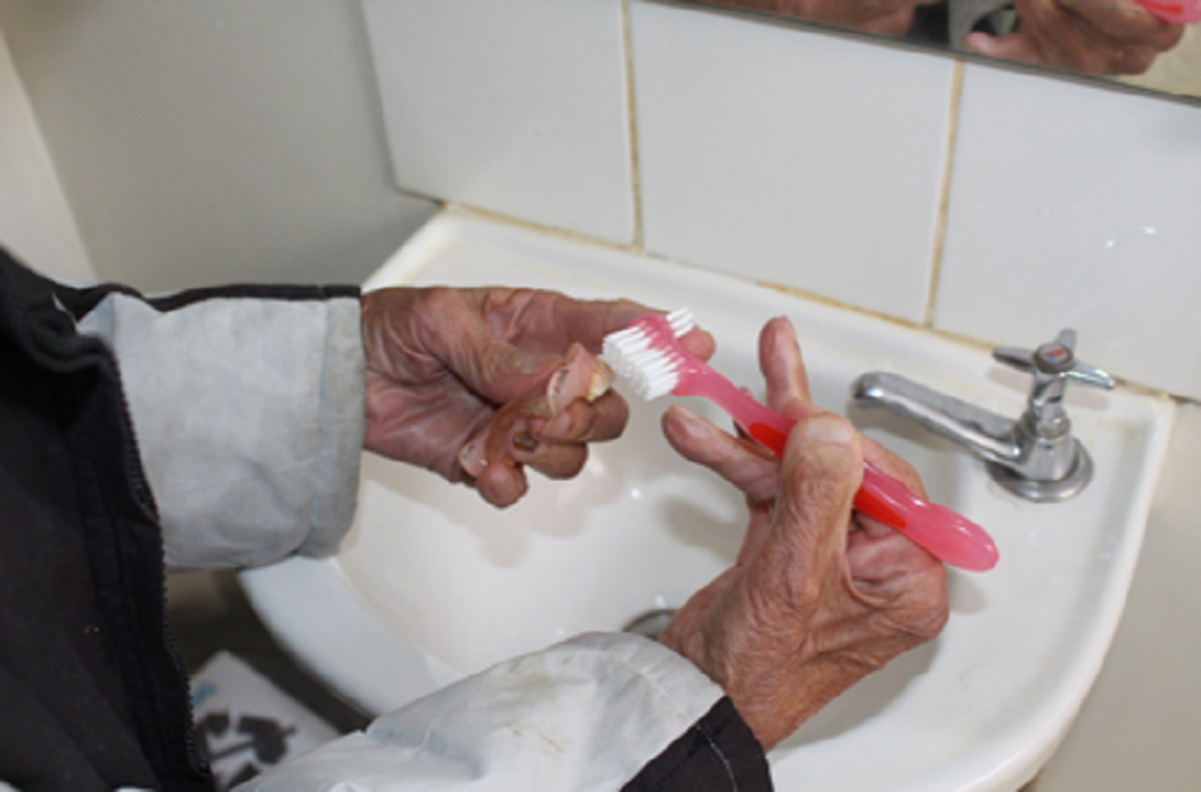
Brush with thickened diameter.

## Discussion

This study has shown that providing leprosy patients with assistive devices increased their independence in oral hygiene activities. Most the patients who accepted and adopted a personalized assistive device showed improved or stable performance of denture brushing or mouthwash. Previous study with leprosy patients showed that to provide devices to them, that allow the care of the self, can contribute to preservation of the independence [18,19] and social inclusion [18]. It was observed too that the use of the devices has an impact in the rescue of the abilities lost with the evolution of the leprosy disabilities [18].

A personalized approach to provision of assistive technology devices was adopted in this study [28], with devices being constructed at low cost to meet the specific needs of patients. The planning took into account the patient’s wishes, the characteristics of their living environment and other contextual factors that would affect case management e.g. whether the patient was living at home with his or her own bathroom or sharing a bathroom in a long-term care facility. Collecting accurate information about each patient’s physical disabilities caused by leprosy made it possible to draw up individual care plans for this group of elders. The main reason for not adherence to intervention was the presence of visual and/or cognitive impairment showing that, although all the patients had a similar history of leprosy, their functional limitations were different and hence they had different needs, requiring different adaptations. In these cases, the intervention should include guidelines and collective work with caregivers, seeking a supportive environment. Comprehensive caregiver orientation and training on device-use should be developed and offered to the elders and their caregivers. Therapists should try to involve caregivers in the treatment and this involvement was previously associated with user satisfaction with device use [29].

Some patients have refused to participate of the study. Some elders related that is easier to perform the relevant tasks without their device or had difficulty incorporating something new into their routine. Previous studies have identified several reasons why older people are reluctant to use assistive technology devices: frustration at having to use them, because the need for them is an indication of their limitations, lack of interest in learning how to use them, poor aesthetic quality, shame and denial of disability [18, 30–35]. In our study the patients’ failure to use the devices provided for them may also be attributed to the fact that many of them feel tired as a consequence of their diseases and the functional limitations caused by their physical deformities and did not want to worry about inserting another new activity or process into their daily life.

The study also demonstrated the importance of multiprofessional working. Collaboration between Dentist and Occupational Therapist was important to development of an assistive device that was appropriate to each patient’s needs [36]. The aim of this intervention was to rehabilitate the patients who were totally or partially dependent on others for denture brushing and mouthwash, in order to enable them to regain or retain their independence in oral self-care for as long as possible.

The sample was a convenience sample of a specific elderly population resident in a former leprosy colony with access to rehabilitation and health-care, so our findings cannot be extended to elders without access to health-care and rehabilitation. The use of a small convenience sample significantly affects the generalizability of the findings, but, we included all elders that need for rehabilitation to oral hygiene. The main strength of the study was the development of simple interventions, planned by the professional team based on patients’ individual needs. This approach highlights the importance of involving leprosy patients in the care process in order to achieve a better oral health and consequently better quality of life. Although this project involved a very specific sample, namely patients whose functional capacity was compromised by the sequelae of leprosy, the approach used in the Casa de Saúde Santa Izabel could be replicated with leprosy patients in other Brazilian ex-colonies. It could also be used as a guide to rehabilitation for other patient groups with impaired manual dexterity due to other conditions, such as rheumatoid arthritis, or patients with tetraplegia, mental or physical impairments, muscular dystrophy and common symptoms aging [36, 37].

This study demonstrated that assistive technology devices could facilitate oral hygiene activities in leprosy patients. It also reinforces the importance of using a multidisciplinary team to provide rehabilitation for this group.

## Supporting information

S1 File Database. (XLSX)
